# Vascular access conversion and patient outcome after hemodialysis initiation with a nonfunctional arteriovenous access: a prospective registry-based study

**DOI:** 10.1186/s12882-017-0492-y

**Published:** 2017-02-22

**Authors:** Natalia Alencar de Pinho, Raphael Coscas, Marie Metzger, Michel Labeeuw, Carole Ayav, Christian Jacquelinet, Ziad A Massy, Bénédicte Stengel, on behalf of the French REIN registry

**Affiliations:** 1 Paris Saclay University, Paris-Sud Univ, UVSQ, CESP, INSERM, Renal and Cardiovascular Epidemiology Team, Villejuif, France; 2 Division of Vascular Surgery, Ambroise Paré University Hospital, Boulogne-Billancourt, France; 3 Lyon-Sud University Hospital, Pierre-Bénite, France; 4 CHRU Nancy, Pôle S2R, Epidémiologie et Evaluations Cliniques, Nancy, France; 5 Inserm, CIC-1433 Epidémiologie Clinique, Nancy, France; 6 Biomedicine Agency, Saint Denis, France; 7 Division of Nephrology, Ambroise Paré University Hospital, Boulogne-Billancourt, France

**Keywords:** Arteriovenous fistula, Catheter, Chronic hemodialysis, Epidemiology, Vascular access

## Abstract

**Background:**

Little is known about vascular access conversion and outcomes for patients starting hemodialysis with nonfunctional arteriovenous (AV) access. We assessed mortality risk associated with nonfunctional AV access at hemodialysis initiation, taking subsequent changes in vascular access into account.

**Methods:**

We studied the 53,092 incident adult hemodialysis patients included in the French REIN registry from 2005 through 2012. AV access placed predialysis was considered nonfunctional when dialysis began with a central venous catheter. Information about vascular access changes was obtained from treatment modality updates.

**Results:**

At hemodialysis initiation, AV access was functional for 47% of patients and nonfunctional for 9%; 44% had a catheter alone. After a 3-year follow-up, 63% of patients beginning hemodialysis with a nonfunctional AV access had changed to a functional one, 4% had had a transplant, 19% had died before any vascular access change, and 13% still used a catheter. Cox proportional hazard models with vascular access treated as a time-dependent variable showed an adjusted mortality hazard ratio (95% confidence interval) for patients with nonfunctional AV access who subsequently converted to functional access of 0.95 (95% CI 0.89–1.03) compared with the reference group with functional AV access since first hemodialysis, versus 1.43 (95% CI 1.31–1.55) for those who did not convert.

**Conclusions:**

Among patients starting hemodialysis with a nonfunctional AV access, a substantial percentage may never experience successful vascular access conversion. Poor survival seems to be limited to these patients, while those who subsequently convert to functional AV access have similar mortality risk compared to patients with such access since hemodialysis initiation. Every effort should be made to obtain functional AV access in all suitable patients.

**Electronic supplementary material:**

The online version of this article (doi:10.1186/s12882-017-0492-y) contains supplementary material, which is available to authorized users.

## Background

Survival of hemodialysis patients is strongly related to the type of vascular access. Numerous studies have shown that arteriovenous (AV) access (either fistulae or grafts) is associated with lower mortality [1–4] and fewer morbid events [5, 6] than central venous catheters. Guidelines for vascular access agree that AV access is the best option for hemodialysis patients, but there is no consensus about the optimal timing for creation, especially for AV fistulae [7–9]. Hence, AV access use at hemodialysis initiation does not come close to meeting current therapeutic goals [10]. Only 18% of US patients start hemodialysis with functional AV access, and this rate does not exceed 30 to 45% in Europe [2, 3, 11, 12].

The lack of functionality of a significant number of AV fistulae and grafts created before hemodialysis initiation results in initial catheter use. Nonfunctionality rates of about 18% are reported among the overall populations of incident hemodialysis patients in Canada and the US [2–4]. Rates as high as 45% have been observed in elderly patients with predialysis AV fistula creation [13]. Because of differences in patient selection and practices for AV access placement [14–16], nonfunctionality rates, their determinants, and outcomes may differ between Europe and North America. Only a few studies have investigated outcomes when AV access was nonfunctional at hemodialysis initiation, all of them in North America [2, 3, 13]. They report that survival is poorer with a nonfunctional than functional AV access and worst with catheters alone [2, 3]. High rates of vascular access conversion in patients starting dialysis with a nonfunctional AV access may explain their better outcome, as suggested by studies in patients who started with a catheter and subsequently converted to a functional AV access [17–19]. Nonetheless, considering outcomes specifically after hemodialysis initiation with nonfunctional AV access is important in the context of the current *Fistula First Catheter Last* initiative, which seeks to increase the use of AV fistulae in patients in whom they are deemed feasible [20]. We therefore used data from the French Renal Epidemiology and Information Network (REIN) registry to study changes of vascular access in patients starting hemodialysis with a nonfunctional AV access and the impact on outcome in a setting with relatively high AV access use.

## Methods

### Population

**Fig. 1:**
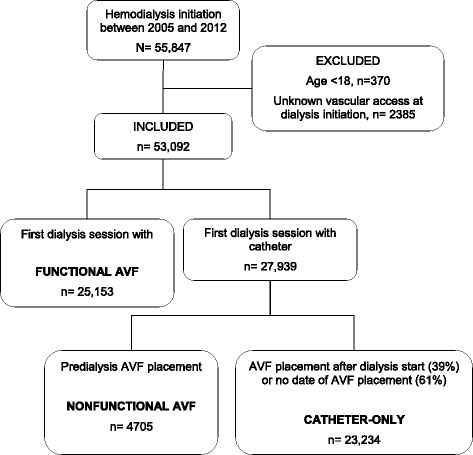
Cohort selection. *Abbreviation*: *AV* arteriovenous

The French REIN registry includes all patients on renal replacement therapy (RRT) for end-stage renal disease (ESRD) – either dialysis or transplantation. It began in 2002 and progressively expanded to include the entire country in 2011. Details on methods and quality control of the REIN registry have been described elsewhere [21]. In this study, we considered the 55,847 patients who started hemodialysis from 2005 through 2012. After excluding minors (*n* = 370) and patients with missing vascular access data (*n* = 2385), we classified the remaining 53,092 patients according to their vascular access at hemodialysis initiation and by 2 data items (Fig. 1): 1/“indicate if the vascular access at the first dialysis session was a catheter”: “yes/no”; 2/“date of first AV access placement, even if it is not functional at dialysis initiation”: “day/month/year”. When the vascular access at the first hemodialysis session was not a catheter, patients were classified as having functional AV access, regardless of reported creation date (*n* = 25,153, with missing dates for 3855). The remaining 27,939 patients who started hemodialysis with a catheter were divided into 2 groups according to the date of AV access creation: when the date preceded hemodialysis initiation, patients were considered to have nonfunctional AV access (*n* = 4705); if the date was after hemodialysis initiation (9030) or was not reported (14,204), patients were considered to have started hemodialysis with a catheter alone (*n* = 23,234). AV fistulae and AV grafts cannot be distinguished at hemodialysis initiation, but in 2013 only 3% of prevalent patients in France had grafts [12].

### Outcomes

Patients were followed up for mortality, kidney transplantation, peritoneal dialysis switch, and dialysis weaning until December 31, 2013. Information about vascular access changes was obtained from modality treatment updates or annual updates in the REIN registry, which report any permanent change (excluding one-off changes) and specify the type of AV access (fistulae or grafts).

### Statistical analyses

Patient characteristics at baseline were described and compared between 3 groups defined by the type of vascular access at hemodialysis initiation, as described above: functional AV access, nonfunctional AV access, and catheter alone. Because of missing data for some variables (Table 1), we performed multiple imputation of 20 datasets with the fully conditional specification method [22, 23]. The imputation model included all variables in Table 1, as well as geographic region, year of first ESRD treatment, and vital status at the end of follow-up. Analyses through the 20 complete datasets were combined according to Rubin and Schencker’s rules. We studied crude survival with the Kaplan-Meier method and compared the 3 vascular access groups by the log-rank test. We then used the cumulative incident function, with Gray’s test, to estimate rates of conversion to functional AV access over 3 years, as well as those of renal transplantation, peritoneal dialysis switch, dialysis weaning, or death (whichever came first) as competing events, in patients who began hemodialysis with nonfunctional AV access or catheter alone [24]. We also estimated adjusted odds ratios for lack of vascular access conversion associated with patients’ characteristics in these two subgroups. Finally, we used Cox proportional hazard models to estimate crude and sequentially adjusted cause-specific mortality hazard ratios (HRs) and 95% confidence intervals (95%CI) associated with nonfunctional AV access (or catheter alone) at baseline, compared with functional AV access. HRs were first adjusted for demographic variables (age, gender, year of RRT initiation, and geographic region), then for clinical variables (comorbidities and laboratory values over the 1-month period before RRT), and finally for predialysis treatment with erythropoiesis-stimulating agents, dialysis start condition (planned or unplanned), and facility characteristics. A final model included vascular access as a time-dependent variable, which means that, for each death, the Cox model compared the current vascular access of patient(s) who died at time t to that of all other patients who were at risk, i.e., alive on hemodialysis at that time [25].

**Table 1:** Patient characteristics according to vascular access at hemodialysis initiation

Characteristics	Functional AV access	Nonfunctional AV access	Catheter only	Imputed missing data (%)
	*n* = 25,153	*n* = 4705	*n* = 23,234	
Men	65.0	58.2	62.0	0
Age (years, median (IQR))	70.4 (58.2–78.6)	70.6 (59.6–78.9)	72.0 (59.6–80.3)	0
Primary renal disease				
Hypertensive/Vascular	27.3	26.5	25.4	
Diabetic nephropathy	23.1	30.9	21.4	
Glomerulonephritis	12.0	9.1	9.6	0
Polycystic kidney disease	9.7	4.5	2.3	
Other	16.6	17.9	26.2	
Unknown	11.2	11.1	15.3	
Diabetes	38.5	49.0	39.8	1.5
Number of cardiovascular comorbidities				
0	48.8	40.1	40.9	
1	25.5	26.3	24.6	
2	15.1	17.5	18.4	5.8
3	7.5	10.9	11.0	
4 or 5	3.1	5.2	5.2	
Lower limb amputation	2.3	4.3	4.3	5.6
Malignancy	8.2	9.5	15.1	3.1
Mobility status				
Autonomous	87.1	79.5	72.2	
Needs assistance	9.9	14.9	18.8	13.6
Totally dependent	3.0	5.7	9.1	
Body mass index (kg/m^2^)				
<18.5	4.7	5.5	8.0	
[18.5–25.0]	40.9	38.4	44.6	26.4
[25.0–30.0]	32.5	30.6	28.9	
≥30.0	21.9	25.5	18.4	
Serum albumin (g/l, mean ± SD)	35.0 ± 5.9	32.5 ± 6.2	30.9 ± 6.4	43.9
Hemoglobin (g/dl, mean ± SD)	10.6 ± 1.6	10.1 ± 1.7	9.7 ± 1.8	21.0
Predialysis ESA treatment	59.2	55.0	31.1	9.7
Estimated glomerular filtration rate (MDRD ml/min/1.73 m^2^)				
eGFR≤5	8.7	13.2	17.7	
5<eGFR≤10	53.9	52.8	47.4	
10<eGFR≤15	28.9	25.3	23.9	17.4
15<eGFR≤20	6.7	6.4	7.3	
eGFR>20	1.8	2.3	3.6	
Unplanned dialysis start	9.5	37.7	56.9	2.8
Facility type				
In center	92.0	96.9	98.3	
Satellite unit	4.0	1.5	1.0	0
Self-dialysis	4.0	1.6	0.7	
Facility ownership				
Public university	17.9	27.3	30.9	
Public non-university	29.8	31.7	31.6	
Private for-profit	34.0	30.3	28.8	0
Private not-for-profit	18.4	10.7	8.8	

*Abbreviations*: *AV* arteriovenous, *CI* confidence interval, *IQR* interquartile range, *SD* standard deviation, *ESA* erythropoiesis-stimulating agents, *MDRD* Modification of Diet in Renal Disease. *P* values of the comparisons between the 3 groups were statistically significant at <0.0001 for every characteristic in Table 1

Because changes in vascular access were not dated, but reported within update intervals, we hypothesized that they occurred at the midpoint of each update interval. We also restricted analysis to patients followed up at least 3 months because of the uncertainty about vascular access change reports in patients dying within the first 3 months of hemodialysis. Mortality rates per 1000 person-years were thus calculated considering the beginning of follow-up at 3 months. Conversions to functional AV access within the first 3 months were however taken into account for patients included in this analysis. We carried out 2 sensitivity analyses: one using the end instead of the midpoint of each update interval for vascular access, and other limiting the analysis to patients with at least one annual update (84% of the study population). The proportional hazard assumption was assessed by plotting scaled Schoenfeld residuals versus rank time. Log-linearity was assessed for continuous variables. Two-sided significance tests were used and *P*-values <0.05 were considered significant. Robust variance estimates were used to account for facility clustering effects. All statistical analyses were performed with SAS 9.4 (SAS Institute Inc, Cary, NC).

## Results

Patients’ median age was 71 (IQR, 59–79) years, 63% were men, 40% had diabetes and 30% at least 2 cardiovascular comorbidities. Dialysis start was unplanned for 33%. Overall, 44% started dialysis with a catheter alone, and 56% after predialysis AV access placement: 47% were functional and 9% nonfunctional. The percentage of patients with predialysis AV access placement decreased significantly from 60% in 2005 to 54% in 2012 (*P*-value for trend <0.001) while that of nonfunctional AV access remained stable. Table 1 summarizes patient characteristics according to vascular access type at the start of hemodialysis.

### Changes in vascular access

**Fig. 2:**
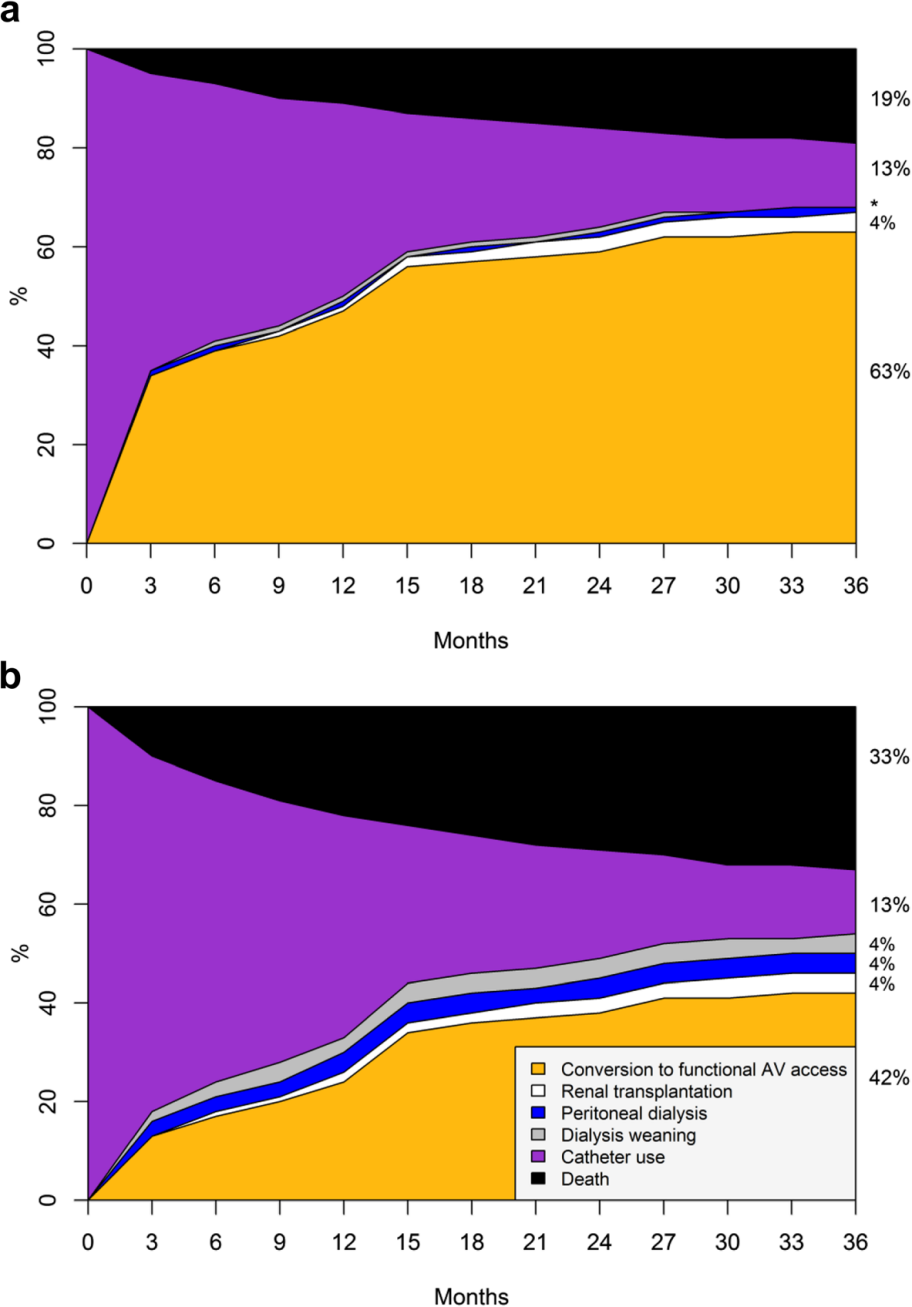
Cumulative incidence^#^ of vascular access conversion to functional AV access. (**a**) Nonfunctional AV access group and (**b**) Catheter only group at hemodialysis initiation. ^#^Competing risks considered were renal transplantation, peritoneal dialysis switch, dialysis weaning, and death, whichever came first. Gray’s test *P*-value (nonfunctional AV access versus catheter only): conversion to functional AV access, peritoneal dialysis switch, dialysis weaning, and death, <0.0001; renal transplantation, 0.04. *Abbreviation*: *AV* arteriovenous. *Cumulative incidence of peritoneal dialysis and dialysis weaning in the nonfunctional AV access group: 0.8 and 0.7%, respectively

The median follow-up was 3 years (range 1–9 years). Most patients (96%) had updated vascular access information in the first 3 months of hemodialysis; 91, 88, and 84% had updated information in the first, second, and third year of follow-up, respectively. Overall, 75% of patients alive on hemodialysis at 12 months had functional AV access, 81% at 24 months, and 84% at 36 months. These percentages were lower for patients with nonfunctional AV access at baseline than for those with functional AV access, but higher than for patients with catheter alone (Additional file 1). Of note, between 2 and 3% of patients classified with functional AVF actually had a functional AV graft at these time points, with no significant difference among groups. At the end of follow-up, 3075 (65.4%) and 10,156 (43.7%) patients in the nonfunctional AV access group and catheter-only group, respectively, have had at least one conversion to functional AV access. The cumulative incidence of vascular access conversion, with death and renal transplantation treated as competing events, was higher in patients with nonfunctional AV access at hemodialysis initiation than in those with catheters alone. Patients with catheter alone at dialysis initiation were nearly twice as likely to die before any reported conversion to functional AV access as those starting dialysis with nonfunctional AV access (Fig. 2a and b). Factors associated with lack of conversion to functional AV access in patients with either nonfunctional AV access or catheter only at baseline are shown in Table 2. Overall, patients who did not convert to a functional AV access were older, most often women, and had poorer health condition. However, the effect of age, malignancy, and poor mobility status on the lack of access conversion appeared to be stronger in patients with catheter only as compared to those with nonfunctional AV access at baseline.

**Table 2:** Patient characteristics associated with lack of conversion to functional AV access in patients starting hemodialysis with nonfunctional AV access or with catheter only

Characteristics	Vascular access at baseline				*P*-value for interaction^a^
	Nonfunctional AV access		Catheter only		
	aOR (95% CI)	*P*-value	aOR (95% CI)	*P*-value	
Women	1.25 (1.10–1.43)	0.0009	1.28 (1.20–1.36)	<0.0001	0.9514
Age (1-year increase)	1.01 (1.01–1.02)	<0.0001	1.02 (1.01–1.02)	<0.0001	0.0117
Primary renal disease		0.5615		<0.0001	0.0938
Hypertensive/Vascular	0.94 (0.73–1.22)		0.93 (0.83–1.04)		
Diabetic nephropathy	0.94 (0.71–1.25)		0.84 (0.74–0.95)		
Glomerulonephritis					
Polycystic kidney disease	1.11 (0.76–1.61)		0.79 (0.64–0.97)		
Other	1.13 (0.87–1.47)		1.19 (1.07–1.33)		
Unknown	1.00 (0.75–1.34)		1.15 (1.03–1.29)		
Diabetes	0.92 (0.77–1.10)	0.3405	0.99 (0.92–1.07)	0.8856	0.6441
Number of cardiovascular comorbidities		0.0008		<0.0001	0.4444
0					
1	1.19 (1.01–1.40)		1.12 (1.04–1.21)		
2	1.27 (1.04–1.54)		1.39 (1.28–1.52)		
3	1.45 (1.16–1.82)		1.58 (1.42–1.75)		
4 or 5	2.09 (1.55–2.82)		1.88 (1.63–2.17)		
Lower limb amputation	1.13 (0.73–1.74)	0.5884	1.26 (1.07–1.47)	0.0050	0.3740
Malignancy	1.23 (0.99–1.52)	0.0592	1.65 (1.51–1.79)	<0.0001	0.0056
Mobility status		<0.0001		<0.0001	0.0105
Autonomous					
Needs assistance	1.41 (1.17–1.69)		1.61 (1.48–1.74)		
Totally dependent	2.05 (1.55–2.70)		2.90 (2.56–3.28)		
Body mass index (kg/m^2^)		0.9333		0.0003	0.0856
<18.5	1.03 (0.73–1.47)		1.21 (1.07–1.36)		
[18.5–25.0]					
[25.0–30.0]	1.01 (0.86–1.19)		0.96 (0.89–1.03)		
≥30.0	1.03 (0.86–1.23)		0.85 (0.78–0.94)		
Serum albumin (1-g/l increase)	0.99 (0.98–1.01)	0.3773	0.99 (0.99–1.00)	0.0033	0.2750
Anemia (hemoglobin <10 g/dl)	0.95 (0.83–1.10)	0.5156	0.91 (0.85–0.97)	0.0027	0.6696
Predialysis ESA treatment	1.12 (0.98–1.27)	0.0899	1.04 (0.97–1.11)	0.3031	0.3383
Estimated glomerular filtration rate (MDRD ml/min/1.73 m^2^)		0.0037		<0.0001	0.9829
eGFR≤5	0.68 (0.54–0.86)		0.75 (0.68–0.83)		
5<eGFR≤10	0.88 (0.74–1.03)		0.91 (0.84–0.99)		
10<eGFR≤15					
15<eGFR≤20	1.11 (0.83–1.48)		1.19 (1.04–1.37)		
eGFR>20	1.38 (0.90–2.12)		1.35 (1.13–1.60)		
Facility type		0.0001		0.0001	0.3805
In center					
Satellite unit	0.37 (0.19–0.73)		0.59 (0.42–0.82)		
Self-dialysis	0.28 (0.14–0.57)		0.48 (0.35–0.65)		
Facility ownership		0.0790		0.1452	0.6509
Public university					
Public non-university	0.77 (0.66–0.91)		0.82 (0.76–0.89)		
Private for-profit	0.78 (0.66–0.92)		0.79 (0.73–0.85)		
Private not-for-profit	1.11 (0.88–1.40)		0.94 (0.85–1.05)		

^a^ *P*-value for interaction between patient characteristic and AV access at baseline. ORs were adjusted for year of hemodialysis initiation and for all variables in Table 2. *Abbreviations*: *aOR* adjusted odds ratio, *CI* confidence interval, *AV* arteriovenous, *ref* reference, *ESA* erythropoiesis-stimulating agents, *MDRD* Modification of Diet in Renal Disease

### Mortality associated with nonfunctional AV access

**Fig. 3:**
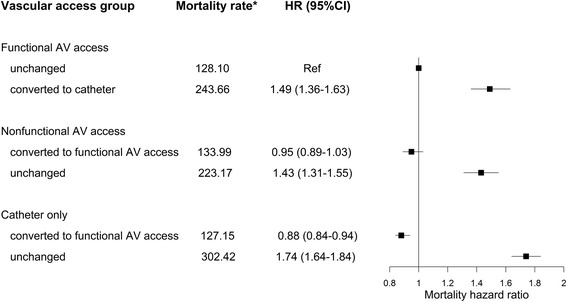
Adjusted mortality hazard ratios associated with baseline vascular access taking subsequent changes into account. Patients with a follow-up shorter than 3 months were excluded. The model was adjusted for all variables in Table 1 as well as region, year of hemodialysis initiation, and vascular access changes as time dependent variable. *Mortality rate per 1000 patient-years. *Abbreviations*: *AV* arteriovenous, *HR* hazard ratio, *CI* confidence interval

During the study period, 22,635 (43%) patients died (of whom 3419 within the first three months of dialysis), 7935 (15%) had kidney transplantation, and 19,100 (36%) remained on hemodialysis. Other outcomes included switch to peritoneal dialysis (3%), dialysis weaning (3%), and loss to follow-up (1%). Kaplan-Meier curves comparing the three vascular access groups at hemodialysis initiation showed survival was best in the functional AV access group, intermediate in the nonfunctional AV access group, and poorest in the catheter alone group (log-rank *P*-value <0.0001; Additional file 2). Cox proportional hazard models showed that the excess risk associated with nonfunctional AV access at hemodialysis initiation was strongly attenuated after adjustment for clinical factors, but remained a significant 10% higher after full adjustment (Additional file 3). The risk of death was lower for patients with nonfunctional AV access than for the catheter alone group. After changes in vascular access were considered, the mortality risk of patients with nonfunctional AV access who subsequently acquired functional access was similar to that of patients whose AV access was functional from the start (Fig. 3). Patients starting dialysis with a catheter alone had the highest risk of death when they continued with a catheter, and the lowest risk if they subsequently acquired a functional AV access. Hazard ratios were stable over time as indicated by the Schoenfeld residual plot (data not shown) Sensitivity analyses using the end instead of the midpoint of each update interval for vascular access or limiting the analysis to patients with at least one annual update did not materially alter these findings (Table 3).

**Table 3:** Sensitivity analyses

Sensitivity analyses :	Setting changes at the end of the interval	Limiting population to those with at least one annual update
Vascular access group	HR (95% CI)	
Functional AV access		
unchanged	1	1
converted to catheter	1.62 (1.48–1.78)	1.60 (1.44–1.77)
Nonfunctional AV access		
converted to functional AV access	1.05 (0.97–1.13)	0.98 (0.91–1.06)
unchanged	1.25 (1.15–1.35)	1.55 (1.41–1.69)
Catheter only		
converted to functional AV access	1.02 (0.96–1.08)	0.90 (0.84–0.95)
unchanged	1.53 (1.45–1.61)	1.94 (1.83–2.06)

Patients with a follow-up shorter than 3 months were excluded. The model was adjusted for all variables in Table 1 as well as region, year of hemodialysis initiation, and vascular access changes as time dependent variable. *Abbreviations*: *AV* arteriovenous, *HR* hazard ratio, *CI* confidence interval

Adjusted mortality hazard ratios associated with initial vascular access taking subsequent changes into account

## Discussion

This study shows that in France, where the rate of predialysis AV access placement is relatively high, nonfunctional AV access at hemodialysis initiation is common and does not appear to have decreased over time. A substantial percentage of patients with nonfunctional AV access at first hemodialysis may never acquire a functional AV access which is associated with increased mortality risk. In contrast, the outcome of patients with a nonfunctional AV access converting to a functional one appears similar to that of those starting with a functional AV access. These findings have important implications for clinical practice and public health policies.

The 9% frequency of nonfunctional AV access at hemodialysis initiation in our study is of the same order of magnitude as that reported in Canada (9%) [4], but lower than in the US (18%) [2, 3]. Nevertheless, the relative weight of nonfunctional AV accesses among all those created predialysis was much lower in France than in North America. About 16% of patients with predialysis AV access started hemodialysis with a catheter in France, while this percentage was 33% in Canada and about 50% in the US. The Dialysis Outcomes and Practice Patterns Study (DOPPS) has pointed out several differences in both patient characteristics and clinical practices between Europe and North America that may explain this discrepancy. For instance, hemodialysis patients in North America have more comorbidities than those in Europe [26], and these are well established determinants of poor AV access use and patency [27, 28]. Most striking, however, are the differences in practices. Not only are rates of AV access at hemodialysis initiation in Europe substantially higher than in North America [14, 29], but surgical training has been shown to differ between these 2 regions, and more training with AV access, as found in Europe, is associated with both AV access creation and patency [30]. Once an AV access is placed, the involvement of the access surgeon in monitoring it for maturation and functional use or in planning for another access in case of failure may impact functionality rates. Nevertheless, information is lacking about the relationship of the access surgeon to the dialysis patient after AV access is placed. Earlier AV fistula cannulation in European countries [14] may also partly explain the lower prevalence of nonfunctional AV access in our study.

As expected, nonfunctional AV access at hemodialysis initiation was associated with an increase of 10% in overall mortality risk, compared with functional AV access. Since AV fistulae account for 97% of the AV access in France, this increased risk is lower than the 30% excess mortality associated with maturing compared with mature AV fistulae observed in all US adult patients who started dialysis between 2006 and 2010 [3]. This excess mortality risk was even higher in elderly patients in the US [2]. Discrepancies in mortality risk estimates associated with nonfunctional AV access at hemodialysis initiation between the 2 countries may, however, reflect differences in the rate of conversion to functional AV access after dialysis initiation, as our findings suggest. The availability of updated vascular access status during follow-up in the REIN registry enabled us to determine that patients with nonfunctional AV access at hemodialysis initiation had lower rates of functional AV access during the first 3 years of RRT than patients who started with functional AV access, but higher rates than patients who started with catheter alone. Our results indicate that the excess mortality risk in patients starting hemodialysis with a catheter, either because no AV access was placed or because it was nonfunctional, is limited to patients who remained on dialysis with a catheter. Thus, once a permanent vascular access was in place for patients with nonfunctional AV access at initiation, their mortality risk was similar to those with functional AV access at first dialysis. Nevertheless, despite adjusting for several potential confounders, we cannot completely rule out a selection effect, with healthier individuals chosen for AV access conversion, as an explanation of the better outcome of this subgroup of patients. Such a selection effect is at least partly indicated by the overall poorer health condition of patients who did not convert to a functional AV access, notably in the catheter only group. Moreover, patients with a catheter alone at baseline and subsequent functional AV access had the lower mortality risk among the studied groups.

These findings, however, are consistent with those from DOPPS, which showed that in patients initiating dialysis with a catheter, conversion to a permanent AV access was associated with an adjusted mortality HR of 0.69 (95% CI, 0.55–0.85) [18]. Similarly, the HEMO study showed no difference in mortality risk between patients with long-term AV access versus those who converted from catheter in the preceding year of treatment [17]. Likewise, in prevalent dialysis patients treated in Fresenius Medical Care facilities, catheter conversion to AV access within a 4-month period was associated with a mortality risk in the following 8 months similar to that of patients with AV access from the outset of the follow-up [19].

Our finding that survival was similar in patients with functional AV access after hemodialysis initiation, regardless of their vascular access at the start, should not be interpreted as calling into question the fistula first principle. In our study, only 63% of patients with nonfunctional AV access at initiation converted to a functional one. Moreover, the catheter use itself may have prevented conversion to a functional access for some patients: catheter use is associated with mortality risk, and some patients may have had catheter-related early deaths. Moreover, catheter use may contribute to subsequent AV access failure due to central venous stenosis [5, 31, 32]. Given that AV fistulae may never be successful for some fraction of patients because of their poor vascular condition, the potential of AV grafts for reducing catheter use should be considered. Studies in the US have shown similar outcomes for AV fistulae and AV grafts in some subpopulations [33, 34].

Major strengths of this study include the size and unselected nature of our registry-based population, which enable generalization of our results to France and other European countries having similar context. In addition, we were able to take major potential confounders into account in the multivariate analyses, including numerous comorbidities and treatment conditions. Lastly, updated information on vascular access was available, and very few patients were lost to follow-up.

Our study also has limitations. First, the prevalence of nonfunctional AV access may be underestimated due to missing AV creation dates. Because the presence of AV access itself at start is not recorded, but only its date of creation if any, we do not know how many patients among those with no reported creation date had no AV access and how many had a missing date for AV access creation. The percentage of missing dates is known, however, for patients who started with functional AV access (15%). Simulation of missing date rates showed that their potential impact on the prevalence estimate of nonfunctional AV access would be low: for example, 10% instead of 9%, if the missing date rate was 15% for both the functional and nonfunctional AV access groups, and 13%, if this rate was twice as high (30%) for the nonfunctional access group. Second, available data did not allow to specify whether the nonfunctional access was patent but not sufficiently matured, or failed and not salvageable. Finally, the vascular access update was interval-censored, which may have resulted in misclassification. Nevertheless, sensitivity analysis using the end of each interval provided consistent findings. Moreover, because annual updates in the REIN registry concern patients’ permanent treatment, our data are likely to reflect outcomes associated with long-term use of a given vascular access.

## Conclusion

In France with its relatively high rate of predialysis AV access creation, AV access is nonfunctional at hemodialysis initiation for a substantial percentage of patients. Importantly, conversion to functional AV access in patients who started on catheter is associated with similar mortality risk as compared to that of patients with functional AV access from the beginning. However, the reasons why a significant number of predialysis AV accesses never become functional require further investigation to identify potentially modifiable risk factors.

## Additional files

Additional file 1: Prevalence of functional AV access at 12, 24, and 36 months according to the type of vascular access at hemodialysis initiation. Abbreviation: AV, arteriovenous. (TIF 1406 kb)
Additional file 2: Kaplan-Meier survival curves according to vascular access group at hemodialysis initiation. Abbreviation: AV, arteriovenous. (TIF 2362 kb)
Additional file 3: Crude and adjusted hazard ratios of mortality according to vascular access at first hemodialysis for patients followed for three months or more. (DOCX 12 kb)

## Abbreviations

AV: Arteriovenous
CI: Confidence interval
ESRD: End-stage renal disease
HR: Hazard ratio
REIN: Renal epidemiology and information network
RRT: Renal replacement therapy
US: United States
