# Supplementary figures and images for: Vascular access conversion and patient outcome after hemodialysis initiation with a nonfunctional arteriovenous access: a prospective registry-based study
Source: BMC Nephrol. 2017 Feb 22;18:74. doi: 10.1186/s12882-017-0492-y (PMC5320699; doi:10.1186/s12882-017-0492-y)

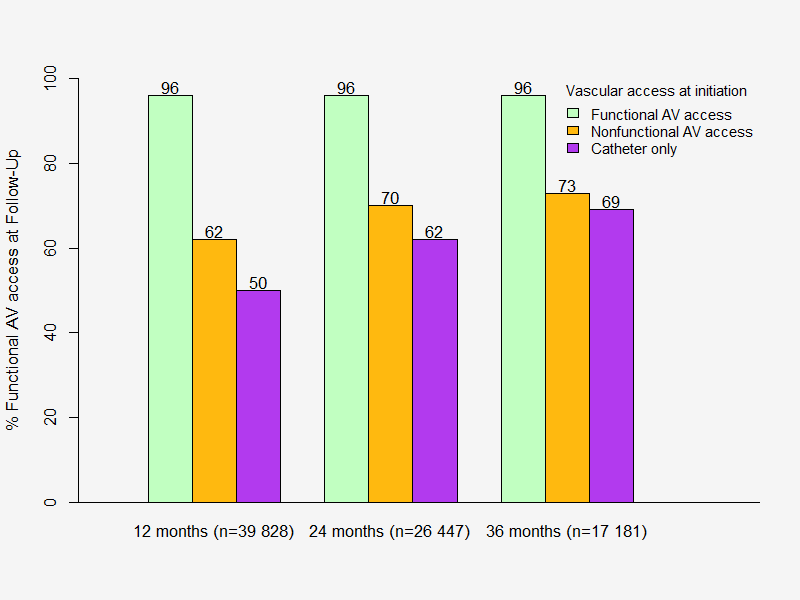

Supplement: Additional file 1: — Prevalence of functional AV access at 12, 24, and 36 months according to the type of vascular access at hemodialysis initiation. Abbreviation: AV, arteriovenous. (TIF 1406 kb) [file 12882_2017_492_MOESM1_ESM.tif]

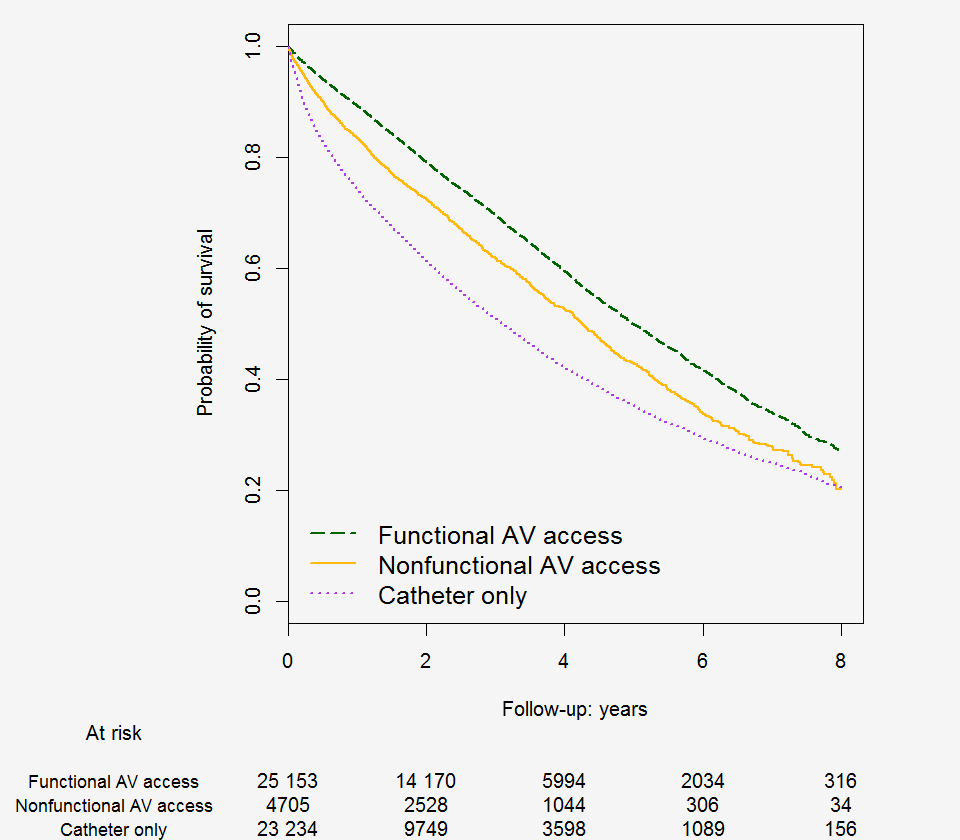

Supplement: Additional file 2: — Kaplan-Meier survival curves according to vascular access group at hemodialysis initiation. Abbreviation: AV, arteriovenous. (TIF 2362 kb) [file 12882_2017_492_MOESM2_ESM.tif]
