# Supplementary material for: Vascular access conversion and patient outcome after hemodialysis initiation with a nonfunctional arteriovenous access: a prospective registry-based study
Source: BMC Nephrol. 2017 Feb 22;18:74. doi: 10.1186/s12882-017-0492-y (PMC5320699; doi:10.1186/s12882-017-0492-y)
Supplement: Additional file 3: — Crude and adjusted hazard ratios of mortality according to vascular access at first hemodialysis for patients followed for three months or more. (DOCX 12 kb) [file 12882_2017_492_MOESM3_ESM.docx]

Table S1. Crude and adjusted hazard ratios of mortality according to vascular access at first hemodialysis for patients followed for three months or more.

| **Vascular access** | **Number of events** | **Crude mortality rate (per 1000 patient-year)** | **HR (95% CI)** |
| --- | --- | --- | --- |
| **Status at hemodialysis initiation** | | | |
| **Functional AV access** | 8453 | 132.62 | 1 |
| **Nonfunctional AV access** | 1819 | 164.05 |  |
| Unadjusted |  |  | 1.24 (1.18-1.31) |
| Model 1 |  |  | 1.30 (1.23-1.37) |
| Model 2 |  |  | 1.12 (1.06-1.19) |
| Model 3 |  |  | 1.10 (1.04-1.17) |
| **Catheter alone** | 8944 | 205.50 |  |
| Unadjusted |  |  | 1.56 (1.49-1.63) |
| Model 1 |  |  | 1.64 (1.58-1.70) |
| Model 2 |  |  | 1.28 (1.23-1.34) |
| Model 3 |  |  | 1.25 (1.20-1.31) |

**Model 1**: adjusted for geographic region, year of hemodialysis initiation, age, and gender. **Model 2**: adjusted for variables in model 1 plus primary renal disease, history of diabetes, number of cardiovascular comorbidities, lower limb amputation, malignancy, mobility, serum albumin level, anemia, body mass index, and estimated glomerular filtration rate. **Model 3**: adjusted for variables in model 2 plus facility type, facility ownership, predialysis erythropoiesis-stimulating agent treatment, and unplannned dialysis start. Abbreviations: AV, arteriovenous; HR, hazard ratio; CI, confidence interval.
